# Supplementary material for: An alternative CTCF isoform antagonizes canonical CTCF occupancy and changes chromatin architecture to promote apoptosis
Source: Nat Commun. 2019 Apr 4;10:1535. doi: 10.1038/s41467-019-08949-w (PMC6449404; doi:10.1038/s41467-019-08949-w)

An alternative CTCF isoform antagonizes canonical CTCF occupancy and changes chromatin architecture to promote apoptosis

Jiao Li, Kaimeng Huang, Gongcheng Hu, Isaac A. Babarinde, Yaoyi Li, Xiaotao Dong, Yu-Sheng Chen, Liping Shang, Wenjing Guo, Junwei Wang, Zhaoming Chen, Andrew P. Hutchins, Yun-Gui Yang, Hongjie Yao

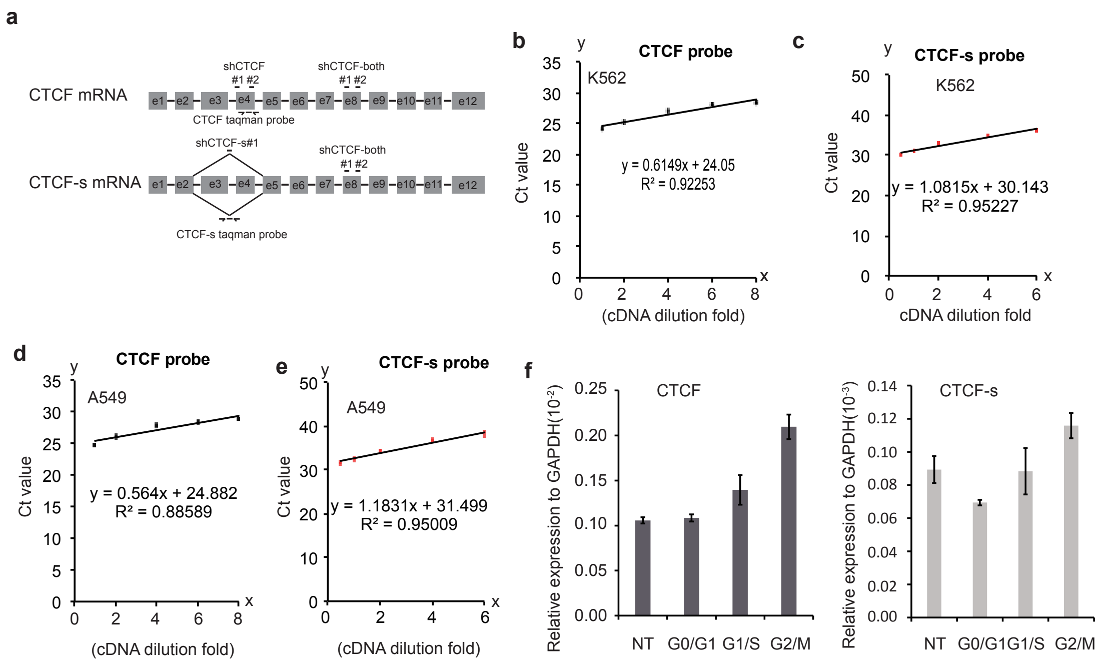

Supplementary Figure 1. Examination of CTCF-s and CTCF expressions in various human cell lines and during cell cycle of HeLa-S3 cells. **a** The schematic diagram of target sites of shRNA and the position of TaqMan probe used for CTCF and CTCF-s. **b-e** Specific TaqMan probes were designed to amplify the CTCF and CTCF-s isoforms. Primer evaluations were performed in cDNA templates from K562 and A549 cells, with different dilutions. Scatterplot showed the linear correlation between the cDNA templates and Ct values. Each dilution was analyzed in duplicate. **f** TaqMan RT-qPCR analysis of the relative expression levels of CTCF and CTCF-s during the cell cycle of HeLa-S3 cells. Source data are provided as a Source Data file.

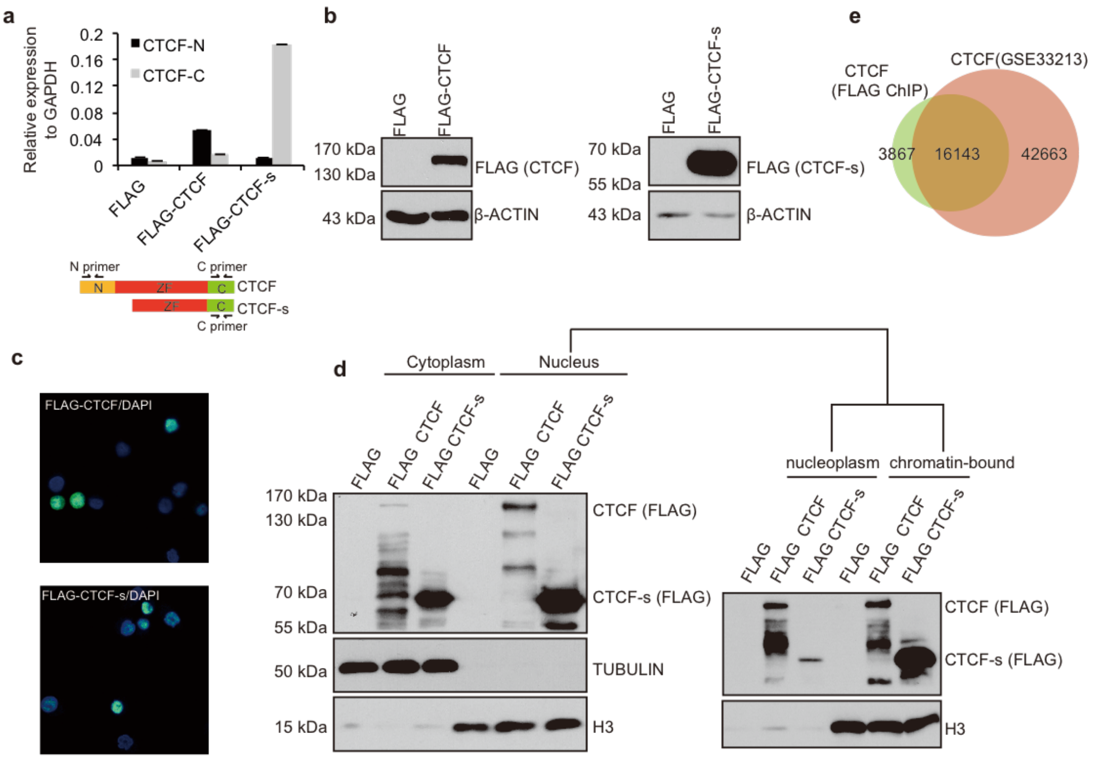

Supplementary Figure 2. Characterization of CTCF and CTCF-s. **a** RT-qPCR analysis to determine the ectopic overexpression level of CTCF and CTCF-s. Primers targeted to the N terminus or C terminus of CTCF were used to verify the overexpression levels. Schematic representation of primer location for detection of CTCF/CTCF-s (bottom). **b** Western blot analysis to determine the overexpression of FLAG-CTCF or FLAG-CTCF-s. **c** The subcellular locations of CTCF and CTCF-s were determined by immunofluorescence assay. FLAG-tagged CTCF or CTCF-s was stably transfected into HeLa-S3 cells. The immunofluorescence was performed with anti-FLAG antibody to show the location of FLAG-CTCF or FLAG-CTCF-s. **d** Subcellular fractionation analysis of FLAG-CTCF and FLAG-CTCF-s. The quality of fractionation procedure was determined by immunoblotting with anti-TUBULIN (Cytoplasm) and anti-H3 (Nucleus) antibodies (Left). Nucleic fraction was further divided into nucleoplasmic and chromatin-bound fractions (Right). The position of FLAG-CTCF and FLAG-CTCF-s was indicated on the right. **e** Venn diagram showing the overlap of FLAG-enriched CTCF peaks with CTCF peaks from ENCODE (GSE33213). Source data are provided as a Source Data file.

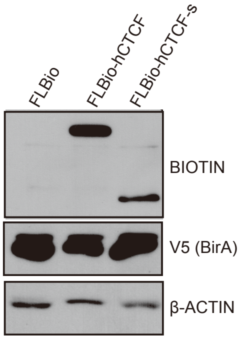

Supplementary Figure 3. Western blot analysis of *in vivo* biotinylation of CTCF and CTCF-s. The antibodies were indicated on the right. Source data are provided as a Source Data file.

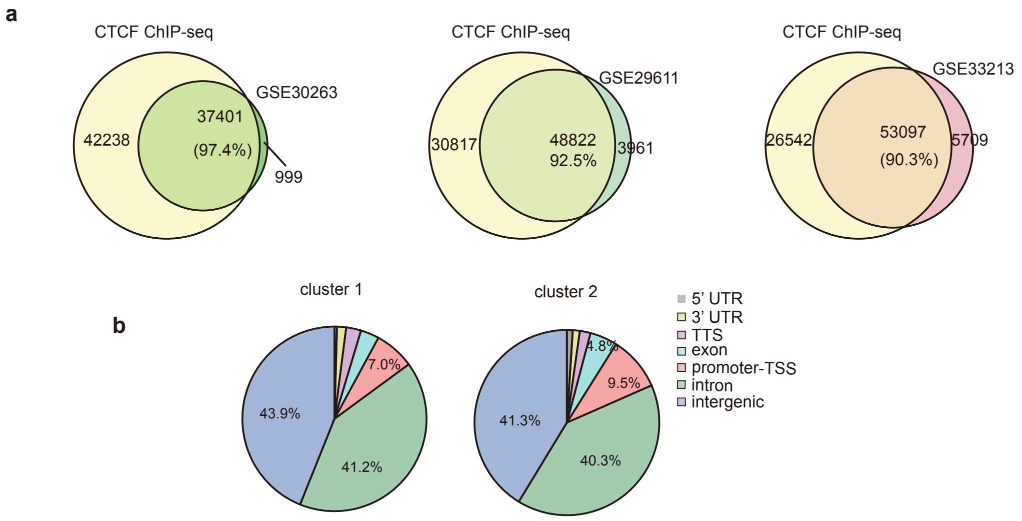

Supplementary Figure 4. CTCF-s competes CTCF at their common peaks. **a** Comparison of our CTCF ChIP-seq data with three CTCF ChIP-seq data from ENCODE. **b** Genomic distribution of CTCF binding peaks from cluster 1 and cluster 2

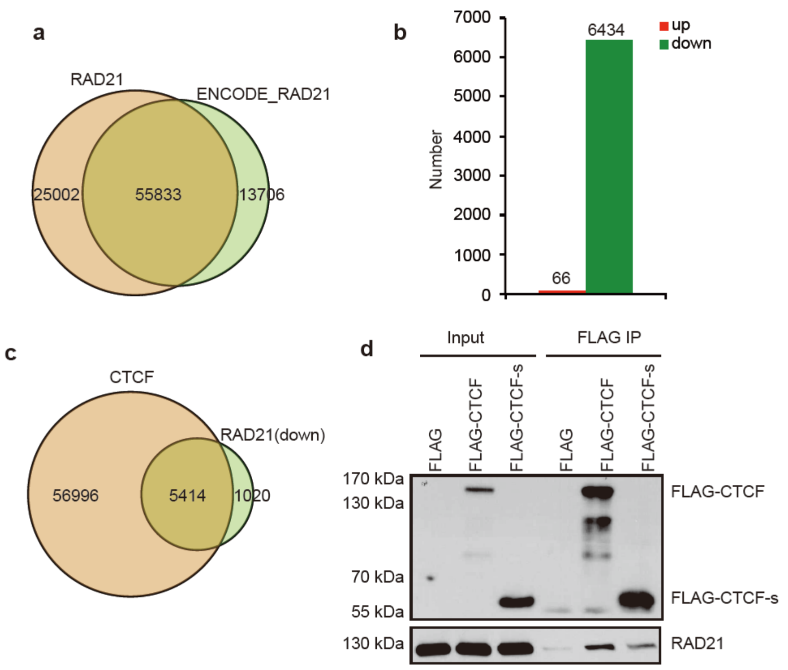

Supplementary Figure 5. Comparison of genome-wide binding between RAD21 and CTCF. **a** Venn diagram showing overlap of RAD21 binding peaks from our ChIP-seq data and ENCODE RAD21 binding sites. **b** Counts of significantly decreased or increased RAD21 binding sites upon CTCF-s gain-of-function. **c** Venn diagram showing the overlap of significantly decreased RAD21 peaks with CTCF peaks. **d** Co-IP analysis of the protein-protein interactions between CTCF/CTCF-s and RAD21. Source data are provided as a Source Data file.

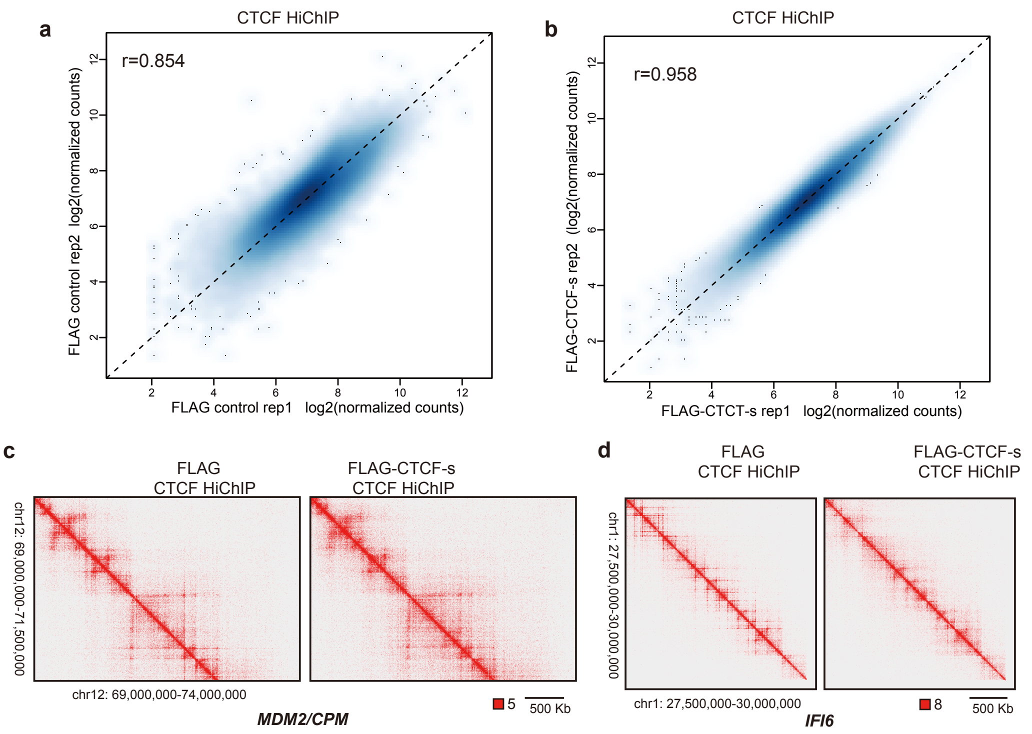

Supplementary Figure 6. Reproducibility analysis of CTCF HiChIP experiments. **a** Reproducibility of loop calling between two independent CTCF HiChiP replicates in FLAG control HeLa-S3 cells. **b** Reproducibility of loop calling between two independent CTCF HiChIP replicates in HeLa-S3 cells after FLAG-CTCF-s gain-of-function. **c** Screenshot of raw interaction map at *MDM2/CPM* loci from FLAG control and FLAG-CTCF-s overexpressing HeLa-S3 cells. Numbers below the interaction maps correspond to maximum signal in the matrix. **d** Screenshot of raw interaction map at *IFI6* loci from FLAG control and FLAG-CTCF-s overexpressing HeLa-S3 cells. Numbers below the interaction maps correspond to maximum signal in the matrix.

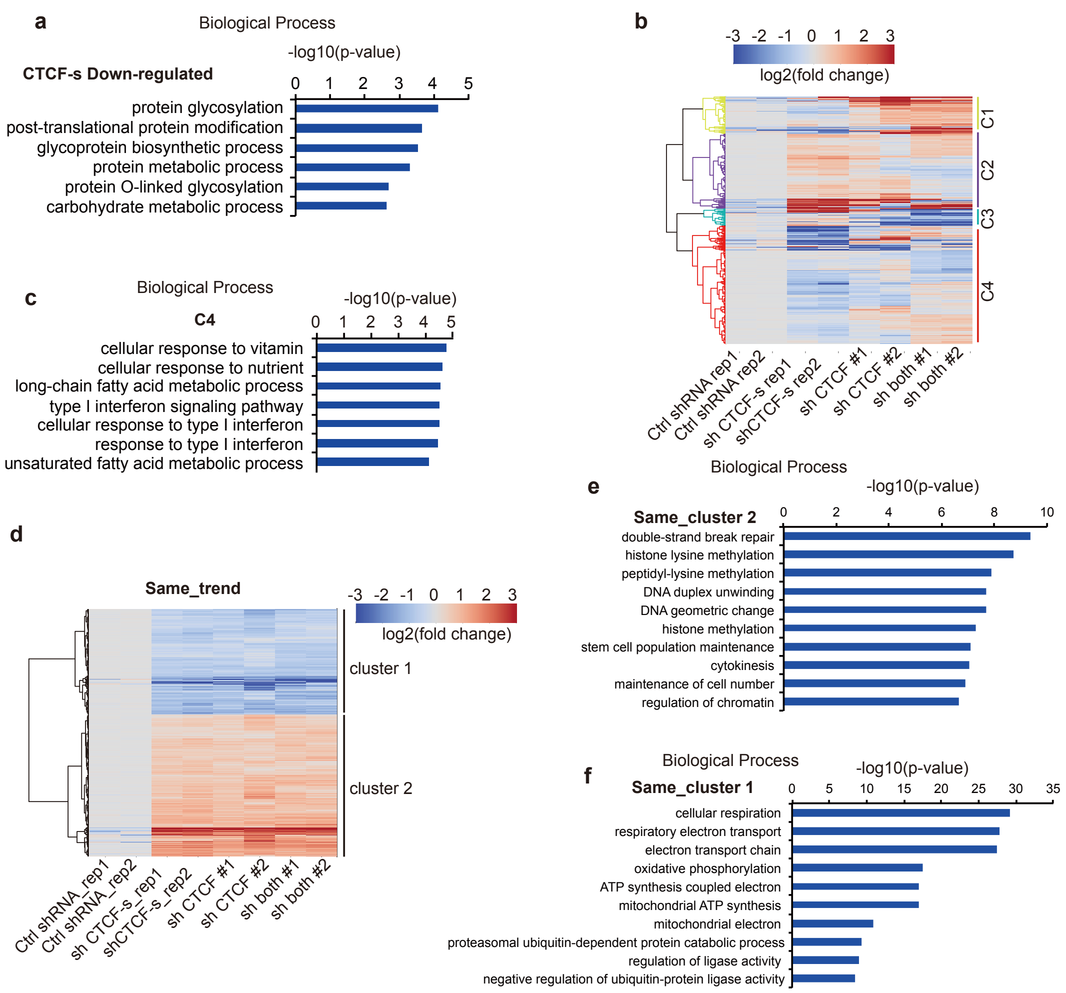

Supplementary Figure 7. GO analysis of DE genes from CTCF-s overexpression or isoform specific shRNA knockdown. **a** GO analysis of the downregulated genes in response to the overexpression of CTCF-s. **b** Hierarchical clustering of DE genes after isoform-specific knockdown in HeLa-S3 cells. For Ctrl shRNA and shRNA targeting to CTCF-s, we used two biological replicates. For shRNA targeting to CTCF and shRNA targeting to both CTCF and CTCF-s, we used two shRNAs targeting to two different sites. **c** GO analysis of C4 class genes in panel **b**. **d** Hierarchical clustering of same trend DE gene profiles after isoform-specific knockdown in HeLa-S3 cells. For Ctrl shRNA and shRNA targeting CTCF-s, we used two biological replicates. For shRNA targeting both CTCF and CTCF-s, we used two shRNAs targeting two different sites. **e** and **f** GO analysis of the differentially expressed genes from cluster 1 (**f**) and cluster 2 (**e**) in panel **d**.

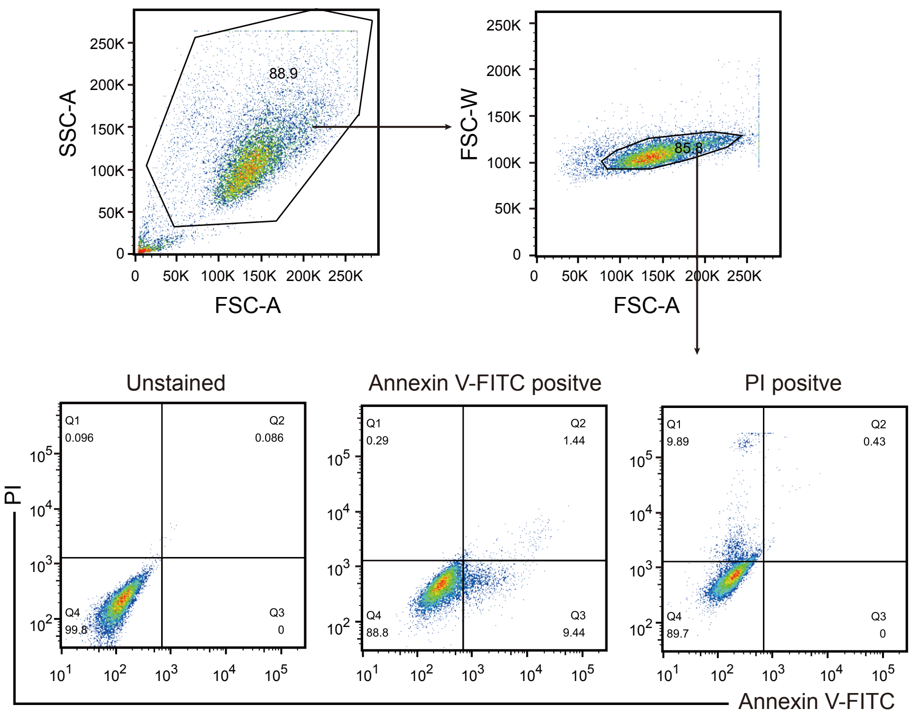

Supplementary Figure 8. Gating strategy for cell apoptosis analysis. We gated the main cell population with FSC-A and SSC-A. Subsequently, we used FSC-A and FSC-W to gate the main single cell population. And Finally, we performed PI and and Annexin V-FITC staining and used unstained sample as negative control, and PI or Annexin V-FITC single-stained cells as positive controls to gate the cell population.

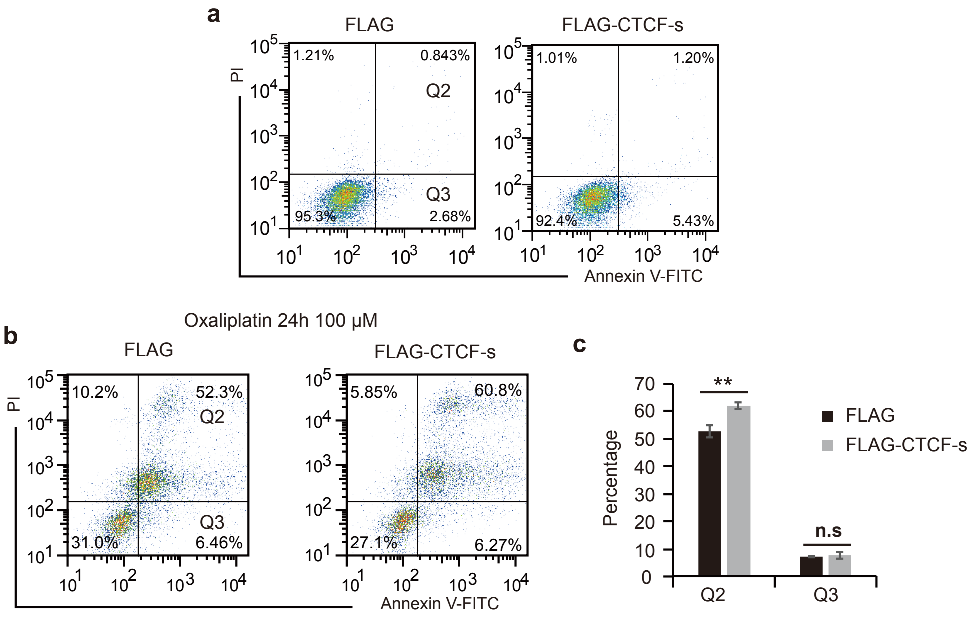

Supplementary Figure 9. CTCF-s promotes cell apoptosis, related to Fig. 5. **a** Apoptosis analysis was determined by Annexin V-FITC/PI staining and FACS analysis after gain-of-function of FLAG, and FLAG-CTCF-s in HeLa-S3 cells, respectively. **b** Apoptosis analysis of FLAG- and FLAG-CTCF-s-overexpressing HeLa-S3 cells following treatment with oxaliplatin for 24 hr. FACS analysis was performed after Annexin V-FITC/PI staining. Plots from one of three independent FACS were shown. **c** Bar chart showing the three biological replicates of apoptosis assay from panel **b**. The data were reported as mean values ± s.d. with the indicated significance by using a two-tailed Student's *t* test (**p<0.01).

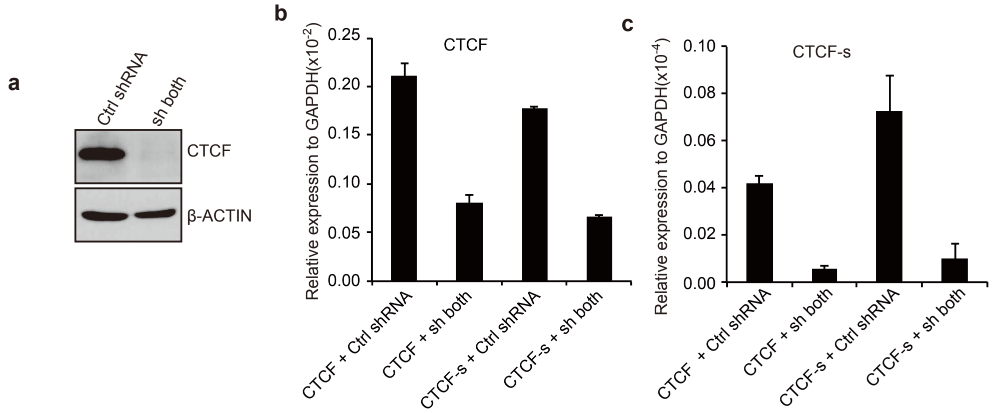

Supplementary Figure 10. Rescue of CTCF or CTCF-s after knocking down endogenous CTCF isoforms, related to Fig. 5. **a** Western blot analysis of endogenous CTCF knockdown efficiency with specific shRNA oligo. **b** TaqMan RT-qPCR analysis of CTCF level after overexpression of either FLAG-CTCF or FLAG-CTCF-s together with endogenous CTCF knockdown. **c** TaqMan RT-qPCR analysis of CTCF-s level after overexpression of either FLAG-CTCF or FLAG-CTCF-s together with endogenous CTCF knockdown. Source data are provided as a Source Data file.

Source Data. Uncropped Western blots and DNA gel

Fig. 1c

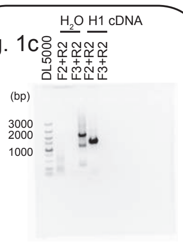

Fig. 1f

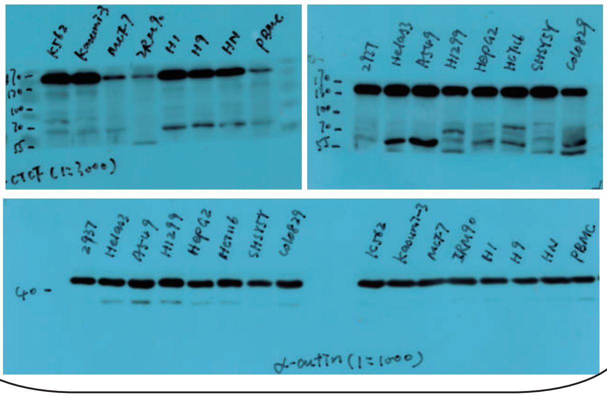

Fig. S2b

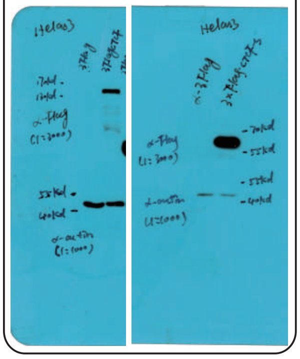

Fig.1h

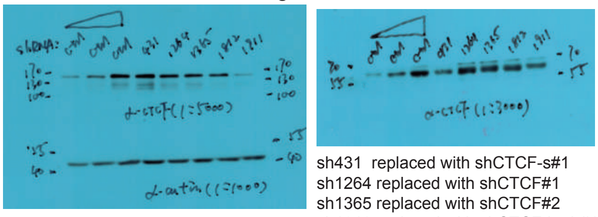

sh431 replaced with shCTCF-s#1
sh1264 replaced with shCTCF#1
sh1365 replaced with shCTCF#2
sh1812 replaced with shCTCF-both#1
sh1911 replaced with shCTCF-both#2

Fig. S2d

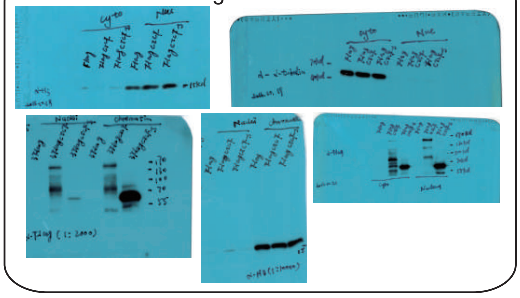

Fig. S5d

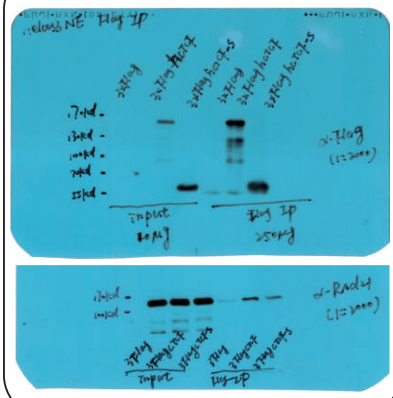

Fig. S3

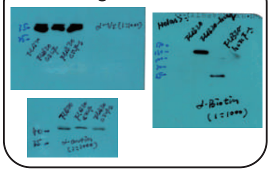

Fig. S10a

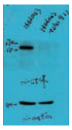

Supplement: Supplementary file 1 — Supplementary Information [file 41467_2019_8949_MOESM1_ESM.pdf]
