# Supplementary material for: An alternative CTCF isoform antagonizes canonical CTCF occupancy and changes chromatin architecture to promote apoptosis
Source: Nat Commun. 2019 Apr 4;10:1535. doi: 10.1038/s41467-019-08949-w (PMC6449404; doi:10.1038/s41467-019-08949-w)
Supplement: Supplementary file 3 — Description of Additional Supplementary Files [file 41467_2019_8949_MOESM3_ESM.pdf]

Description of Additional Supplementary Files

File Name: Supplementary Data 1
Description: shRNA targets used in this study

File Name: Supplementary Data 2
Description: RT-qPCR primers used in this study

File Name: Supplementary Data 3
Description: Antibodies used in this study

File Name: Supplementary Data 4
Description: HiChIP data validation

File Name: Supplementary Data 5
Description: ChIP-qPCR primers used in this study

File Name: Supplementary Data 6
Description: Primers used for 3C experiments
