# Supplementary material for: An alternative CTCF isoform antagonizes canonical CTCF occupancy and changes chromatin architecture to promote apoptosis
Source: Nat Commun. 2019 Apr 4;10:1535. doi: 10.1038/s41467-019-08949-w (PMC6449404; doi:10.1038/s41467-019-08949-w)
Supplement: Supplementary file 10 — Reporting Summary [file 41467_2019_8949_MOESM10_ESM.pdf]

nature research

Corresponding author(s): Hongjie Yao
Last updated by author(s): Jan 23, 2019

Reporting Summary

Nature Research wishes to improve the reproducibility of the work that we publish. This form provides structure for consistency and transparency in reporting. For further information on Nature Research policies, see Authors & Referees and the Editorial Policy Checklist.

Statistics

For all statistical analyses, confirm that the following items are present in the figure legend, table legend, main text, or Methods section.

n/a	Confirmed	
☒	☐	The exact sample size (*n*) for each experimental group/condition, given as a discrete number and unit of measurement
☐	☒	A statement on whether measurements were taken from distinct samples or whether the same sample was measured repeatedly
☐	☒	The statistical test(s) used AND whether they are one- or two-sided<br>*Only common tests should be described solely by name; describe more complex techniques in the Methods section.*
☒	☐	A description of all covariates tested
☒	☐	A description of any assumptions or corrections, such as tests of normality and adjustment for multiple comparisons
☐	☒	A full description of the statistical parameters including central tendency (e.g. means) or other basic estimates (e.g. regression coefficient) AND variation (e.g. standard deviation) or associated estimates of uncertainty (e.g. confidence intervals)
☐	☒	For null hypothesis testing, the test statistic (e.g. *F*, *t*, *r*) with confidence intervals, effect sizes, degrees of freedom and *P* value noted<br>*Give P values as exact values whenever suitable.*
☒	☐	For Bayesian analysis, information on the choice of priors and Markov chain Monte Carlo settings
☒	☐	For hierarchical and complex designs, identification of the appropriate level for tests and full reporting of outcomes
☐	☒	Estimates of effect sizes (e.g. Cohen's *d*, Pearson's *r*), indicating how they were calculated

Our web collection on statistics for biologists contains articles on many of the points above.

Software and code

Policy information about availability of computer code

Data collection	N/A
Data analysis	N/A

For manuscripts utilizing custom algorithms or software that are central to the research but not yet described in published literature, software must be made available to editors/reviewers. We strongly encourage code deposition in a community repository (e.g. GitHub). See the Nature Research guidelines for submitting code & software for further information.

Data

Policy information about availability of data

All manuscripts must include a data availability statement. This statement should provide the following information, where applicable:

- Accession codes, unique identifiers, or web links for publicly available datasets
- A list of figures that have associated raw data
- A description of any restrictions on data availability

Our sequencing data from ChIP-seq, RNA-seq, HiChIP have been deposited in the Gene Expression Ominbus and the accession number NCBI GEO: GSE108869.

Field-specific reporting

Please select the one below that is the best fit for your research. If you are not sure, read the appropriate sections before making your selection.

☒ Life sciences ☐ Behavioural & social sciences ☐ Ecological, evolutionary & environmental sciences

For a reference copy of the document with all sections, see nature.com/documents/nr-reporting-summary-flat.pdf

Life sciences study design

All studies must disclose on these points even when the disclosure is negative.

Sample size	N/A
Data exclusions	No data were excluded from the analyses.
Replication	All the experiments were performed at least two-biological replicates, and each biological replicates were performed several technical replicates. The detailed replication information are provided in the corresponding figure legends.
Randomization	N/A
Blinding	N/A

Reporting for specific materials, systems and methods

We require information from authors about some types of materials, experimental systems and methods used in many studies. Here, indicate whether each material, system or method listed is relevant to your study. If you are not sure if a list item applies to your research, read the appropriate section before selecting a response.

Materials & experimental systems

n/a	Involved in the study	
☐	☒	Antibodies
☐	☒	Eukaryotic cell lines
☒	☐	Palaeontology
☒	☐	Animals and other organisms
☒	☐	Human research participants
☒	☐	Clinical data

Methods

n/a	Involved in the study	
☐	☒	ChIP-seq
☐	☒	Flow cytometry
☒	☐	MRI-based neuroimaging

Antibodies

Antibodies used	Anti-β Actin antibody, abcam, CatLog:ab3280<br>Anti-CTCF antibody, Millipore, CatLog:07-729<br>Anti-CTCF antibody, Active Motif, CatLog:61311<br>Anti-CTCF antibody, our lab, CatLog:NA<br>Anti-FLAG antibody, Sigma, CatLog:F1804<br>Anti-biotin, HRP-linked antibody, Cell Signaling Technology, CatLog:#7075<br>FLAG-M2 Magnetic beads, Sigma, CatLog:M8823<br>Anti-Tubulin antibody, Sigma, CatLog:T6074<br>Anti-RAD21 antibody, abcam, CatLog:ab992<br>Anti-H3 antibody, Cell Signaling Technology, CatLog:#4620
Validation	For those commercial antibodies, the validation statements are provided on the manufacturer's websites, and also the data provided in this manuscript.

Eukaryotic cell lines

Policy information about cell lines

Cell line source(s)	293T cell line, HeLa-S3 cell line.
Authentication	These cell lines were purchased from ATCC.
Mycoplasma contamination	We confirmed that all cell lines used in this study are negative for mycoplasma contamination.
Commonly misidentified lines (See ICLAC register)	N/A

Methodology

Replicates

For HiChIP experiments, we have performed two biological repeats. For ChIP-seq, since ChIP-seq data were highly convincible as the high overlapping ratio with the published ENCODE dataset, therefore, we did not perform biological replicates.

Sequencing depth

For FLAG ChIP-seq
FLAG control, raw reads 32,386,788, high quality unique reads 23,594,792, read length 150 bp, paired-end.
FLAG-CTCF overexpression, raw reads 50,256,287, high quality unique reads 36,468,522 , read length 150 bp, paired-end.
FLAG-CTCF-s overexpression, raw reads 35,551,107, high quality unique reads 26,084,762, read length 150 bp, paired-end.

For CTCF ChIP-seq
FLAG_input, raw reads 67,226,304, high quality unique reads 53,502,527, read length 75 bp, paired-end.
FLAG_CTCF ChIP-seq, raw reads 65,523,895, high quality unique reads 53,118,020, read length 75 bp, paired-end.
FLAG-CTCF-s_input, raw reads 71,073,427, high quality unique reads 57,334,292, read length 75 bp, paired-end.
FLAG-CTCF-s_CTCF ChIP-seq, raw reads 60,420,904, high quality unique reads 47,904,732, read length 75 bp, paired-end.

For RAD21 ChIP-seq
FLAG_input, raw reads 67,226,304, high quality unique reads 54,204,191, read length 75 bp, single-end.
FLAG_CTCF-s_input, raw reads 71,073,427, high quality unique reads 57,875,576, read length 75 bp, single-end.
FLAG_RAD21 ChIP-seq, raw reads 29,358,349, high quality unique reads 19,601,476, read length 75 bp, single-end.
FLAG-CTCF-s_RAD21 ChIP-seq, raw reads 31,950,672, high quality unique reads 24,098,525, read length 75 bp, single-end.

For biotin ChIP-seq
biotin-CTCF_bioChIP-seq, raw reads 26,074,975, high quality unique reads 15,694,355, read length 150 bp, paired-end.
biotin-CTCF-s_bioChIP-seq, raw reads 32,603,177, high quality unique reads 20,600,686, read length 150 bp, paired-end.
biotin_bioChIP-seq, raw reads 31,296,777, high quality unique reads 17,714,320, read length 150 bp, paired-end.

Antibodies

For CTCF ChIP-seq, we used Anti-CTCF antibody purchased from Active Motif (CatLog:61311).
For biotin ChIP-seq, we used streptavidin M-280 dynabeads purchased from Invitrogen (CatLog:11205D).
For RAD21 ChIP-seq, we used Anti-RAD21 antibody purchased from abcam (CatLog:ab992).
For FLAG ChIP-seq, we used FLAG-M2 beads purchased from sigma (CatLog:M8823).

Peak calling parameters

For FLAG ChIP-seq
macs2 callpeak -t FLAG-CTCF.bam -c FLAG.bam -f BAMPE -g hs -n FLAG-CTCF -p 1e-4 –B –SPMR
macs2 callpeak -t FLAG-CTCF-s.bam -c FLAG.bam -f BAMPE -g hs -n FLAG-CTCF-s -p 1e-4 –B --SPMR

For CTCF ChIP-seq,
macs2 callpeak -t FLAG_CTCF_ChIP-seq.bam FLAG_input.bam -f BAMPE -g hs -n FLAG_control_CTCF-peak –B --SPMR
macs2 callpeak -t FLAG-CTCF-s_CTCF_ChIP-seq.bam -c FLAG-CTCF-s_input.bam -f BAMPE -g hs -n FLAG-CTCF-s_CTCF-peak –B --SPMR

For RAD21 ChIP-seq, high quality unique reads were subsampled into 20M reads to call peaks.
macs2 callpeak -t FLAG_RAD21_ChIP-seq_subsample.bed -c FLAG_input_subsample.bed -f BED -n FLAG_RAD21 -g hs -q 0.01 --nomodel --shift 0 --extsize 130 –B --SPMR
macs2 callpeak -t FLAG-CTCF-s_RAD21_subsample.bed -c FLAG-CTCF-s_input_subsample.bed -f BED -n FLAG-CTCF-s_RAD21 -g hs -q 0.01 --nomodel --shift 0 --extsize 144 -B --SPMR

For biotin ChIP-seq,
macs2 callpeak -t biotin-CTCF_bioChiP.bam –c biotin_bioChIP.bam -f BAMPE -n biotin-CTCF -g hs -B --SPMR
macs2 callpeak -t biotin-CTCF-s_bioChIP.bam -c biotin_bioChIP.bam -f BAMPE -n biotin-CTCF-s -g hs -B --SPMR

Data quality

For FLAG ChIP-seq, due to the background noise, we used macs2 parameter "p 1e-4", flag-CTCF peaks is 11,578, FLAG-CTCF-s peaks is 8,890. Of them, few peaks are above 5-fold enrichment.

For CTCF ChIP-seq, we used FDR 1% (macas2, q = 0.01) as cutoff, and we got 74,386 CTCF peaks in FLAG control samples, and got 79,925 CTCF peaks in FLAG-CTCF-s overexpression samples. Of them, peaks above 5-fold enrichment is 69,755, 65,198, respectively.

For RAD21 ChIP-seq, we used FDR 0.01 (macs2, q = 0.01) as cutoff, and we got 80,362 RAD21 peaks in FLAG control samples, 46,469 RAD21 peaks in FLAG-CTCF-s overexpression samples. Of them, 71,018, 43,201 peaks were above 5-fold enrichment, respectively.

For biotin ChIP-seq, we used FDR 5% (macs2, q = 0.05) as cutoff, and we got 72,937 peaks for CTCF, 41,362 peaks for CTCF-s. Of them, 58,378, 20,938 peaks were above 5-fold enrichment, respectively.

Software

FastQC (v0.11.2), Reads qualification.
TrimGalore (v0.4.4), adapter and low quality reads Trimming.
Bowtie2 (v2.2.5) Reads alignment.
samtools (v1.2), high-quality mapped reads selection.
picard tools (v1.90), PCR duplicates removing.
MACS2 (v2.1.0), Peak calling or differential peak analysis.
bedtools (v2.25.0), peak overlapping analysis.

ChIP-seq

Data deposition

☒ Confirm that both raw and final processed data have been deposited in a public database such as GEO.

☒ Confirm that you have deposited or provided access to graph files (e.g. BED files) for the called peaks.

Data access links<br>*May remain private before publication.*	To review GEO accession GSE108869:<br>Go to https://www.ncbi.nlm.nih.gov/geo/query/acc.cgi?acc=GSE108869<br>Enter token sravsksipjstfmz into the box
Files in database submission	GSM2915162 FLAG_FLAG_ChIPSeq<br>GSM2915163 FLAG-CTCF_FLAG_ChIPSeq<br>GSM2915164 FLAG-CTCF-s_FLAG_ChIPSeq<br>GSM2915165 Control_Input<br>GSM2915166 Control_CTCF_ChIPSeq<br>GSM2915167 CTCF-s_OE_Input<br>GSM2915168 CTCF-s_OE_CTCF_ChIPSeq<br>GSM2915169 FLAG_rep1.RNA-Seq<br>GSM2915170 FLAG_rep2.RNA-Seq<br>GSM2915171 FLAG-CTCF_rep1.RNA-Seq<br>GSM2915172 FLAG-CTCF_rep2.RNA-Seq<br>GSM2915173 FLAG-CTCF-s_rep1.RNA-Seq<br>GSM2915174 FLAG-CTCF-s_rep2.RNA-Seq<br>GSM2915175 CtrlshRNA_rep1.RNA-Seq<br>GSM2915176 CtrlshRNA_rep2.RNA-Seq<br>GSM2915177 shCTCF-s_rep1.RNA-Seq<br>GSM2915178 shCTCF-s_rep2.RNA-Seq<br>GSM2915179 shCTCF#1.RNA-Seq<br>GSM2915180 shCTCF#2.RNA-Seq<br>GSM2915181 shboth#1.RNA-Seq<br>GSM2915182 shboth#2.RNA-Seq<br>GSM2974085 Control_CTCF-HiChIP_rep1<br>GSM2974086 Control_CTCF-HiChIP_rep2<br>GSM2974087 CTCF-s_OE_CTCF-HiChIP_rep1<br>GSM2974088 CTCF-s_OE_CTCF-HiChIP_rep2<br>GSM2990425 Control_RAD21_ChIPSeq<br>GSM2990426 CTCF-s_OE_RAD21_ChIPSeq<br>GSM3466331 biotin_alone_biotin_ChIPSeq<br>GSM3466332 biotin-CTCF_OE_biotin_ChIPSeq<br>GSM3466333 biotin-CTCF-s_OE_biotin_ChIPSeq<br><br>GSE108869_CTCF-s_OE_CTCF-HiChIP_combine_allValidPairs.hic.gz<br>GSE108869_CTCF-s_OE_CTCF_ChIPSeq.narrowPeak.gz<br>GSE108869_CTCF-s_OE_CTCF_ChIPSeq_control.bw<br>GSE108869_CTCF-s_OE_CTCF_ChIPSeq_treat.bw<br>GSE108869_CTCF-s_OE_RAD21_ChIPSeq_control_lambda.bw<br>GSE108869_CTCF-s_OE_RAD21_ChIPSeq_peaks.narrowPeak.gz<br>GSE108869_CTCF-s_OE_RAD21_ChIPSeq_treat_pileup.bw<br>GSE108869_CTCF_CTCF-s_OE_RNA-seq.tsv.gz<br>GSE108869_Control_CTCF-HiChIP_combine_allValidPairs.hic.gz<br>GSE108869_Control_CTCF_ChIPSeq.narrowPeak.gz<br>GSE108869_Control_CTCF_ChIPSeq_control.bw<br>GSE108869_Control_CTCF_ChIPSeq_treat.bw<br>GSE108869_Control_RAD21_ChIPSeq_control_lambda.bw<br>GSE108869_Control_RAD21_ChIPSeq_peaks.narrowPeak.gz<br>GSE108869_Control_RAD21_ChIPSeq_treat_pileup.bw<br>GSE108869_FLAG-CTCF-s.narrowPeak.gz<br>GSE108869_FLAG-CTCF-s_control_lambda.bw<br>GSE108869_FLAG-CTCF-s_treat_pileup.bw<br>GSE108869_FLAG-CTCF.narrowPeak.gz<br>GSE108869_FLAG-CTCF_control_lambda.bw<br>GSE108869_FLAG-CTCF_treat_pileup.bw<br>GSE108869_Knockdown_RNA-seq.tsv.gz<br>GGSE108869_biotin-CTCF-s_bioChIP_redup_with_SPMR_peaks.narrowPeak.gz<br>GSE108869_biotin-CTCF-s_sorted_control_lambda.bw<br>GSE108869_biotin-CTCF-s_treat_pileup.bw<br>GSE108869_biotin-CTCF_bioChIP_redup_with_SPMR_peaks.narrowPeak.gz<br>GSE108869_biotin-CTCF_sorted_control_lambda.bw<br>GSE108869_biotin-CTCF_treat_pileup.bw
Genome browser session<br>(e.g. UCSC)	N/A

deeptools (v2.2.4), ChIP-seq heatmap generation.
homer, Peak annotation and de novo motif finding.

Flow Cytometry

Plots

Confirm that:

☒ The axis labels state the marker and fluorochrome used (e.g. CD4-FITC).

☒ The axis scales are clearly visible. Include numbers along axes only for bottom left plot of group (a 'group' is an analysis of identical markers).

☒ All plots are contour plots with outliers or pseudocolor plots.

☒ A numerical value for number of cells or percentage (with statistics) is provided.

Methodology

Sample preparation	Cells were seeded in 6-well plates and harvested at a density of 6 × 105 cells/ml, and then washed twice with ice-cold PBS. Cells were suspended with 100 µl 1 × binding buffer containing 5 µl of Annexin V-FITC and 5 µl of propidium iodide (PI) and incubated for 10 min in dark at room temperature.
Instrument	BD LSRFortessa Cell Analyzer (Becton-Dickinson, CA, USA)
Software	FLowJo software
Cell population abundance	We gated the main cell population and used 104 cells for further apoptosis analysis. As we did not performed cell sorting, therefore we did not determined the purity of the samples.
Gating strategy	We performed PI and and Annexin V-FITC staining and used unstained sample as negative control, and PI or Annexin V-FITC single-stained cells as positive controls to gate the cell population.

☒ Tick this box to confirm that a figure exemplifying the gating strategy is provided in the Supplementary Information.
